# LABEC, the INFN ion beam laboratory of nuclear techniques for environment and cultural heritage

**DOI:** 10.1140/epjp/s13360-021-01411-1

**Published:** 2021-04-30

**Authors:** M. Chiari, S. Barone, A. Bombini, G. Calzolai, L. Carraresi, L. Castelli, C. Czelusniak, M. E. Fedi, N. Gelli, F. Giambi, F. Giardi, L. Giuntini, S. Lagomarsino, L. Liccioli, F. Lucarelli, M. Manetti, M. Massi, A. Mazzinghi, S. Nava, P. Ottanelli, S. Sciortino, C. Ruberto, L. Sodi, F. Taccetti, P. A. Mandò

**Affiliations:** INFN Division of Florence and Department of Physics and Astronomy, University of Florence, via G. Sansone 1, 50019 Sesto Fiorentino, Italy

## Abstract

The LABEC laboratory, the INFN ion beam laboratory of nuclear techniques for environment and cultural heritage, located in the Scientific and Technological Campus of the University of Florence in Sesto Fiorentino, started its operational activities in 2004, after INFN decided in 2001 to provide our applied nuclear physics group with a large laboratory dedicated to applications of accelerator-related analytical techniques, based on a new 3 MV Tandetron accelerator. The new accelerator greatly improved the performance of existing Ion Beam Analysis (IBA) applications (for which we were using since the 1980s an old single-ended Van de Graaff accelerator) and in addition allowed to start a novel activity of Accelerator Mass Spectrometry (AMS), in particular for ^14^C dating. Switching between IBA and AMS operation became very easy and fast, which allowed us high flexibility in programming the activities, mainly focused on studies of cultural heritage and atmospheric aerosol composition, but including also applications to biology, geology, material science and forensics, ion implantation, tests of radiation damage to components, detector performance tests and low-energy nuclear physics. This paper describes the facilities presently available in the LABEC laboratory, their technical features and some success stories of recent applications.

## Introduction

The acronym LABEC means “Laboratorio di tecniche nucleari per l’Ambiente e i BEni Culturali” (in English: “Laboratory of nuclear techniques for Environment and Cultural Heritage”), but applications in several other fields outside physics, as well as supporting activities to basic nuclear or subnuclear physics experiments (e.g. tests of radiation damage to components, or detector performance tests, etc.), are also performed. LABEC has been created by the Italian National Institute of Nuclear Physics (INFN) within its Florence division and is managed in close cooperation with the Department of Physics and Astronomy of the University of Florence, which funded the construction of the building where the laboratory is located. The present formal configuration was established in 2001, but the group that created LABEC had already been actively working since the mid-1980s in the field of accelerator-based applications to various fields. In turn, this previous activity of application of nuclear analytical techniques was “heir” to a very long tradition of the Florence group in basic research in low-energy nuclear physics.

Let’s therefore start with a short excursus on our “origins”.

Since the beginning of the 1970s, a 3 MV Van de Graaff accelerator had been installed in the building of the Department of Physics in Florence, located at those times on the “historical” hills of Arcetri.^1^ This Van de Graaff (KS3000) had been built in 1955 to be the injector of the electrosynchrotron in the INFN National Laboratories of Frascati (Rome): when the electrosynchrotron ended to be used in the late 1960s, the VdG injector was transferred to Florence, completely refurbished and converted by the local group into a positive ion machine (KN3000). As such, it started a second life and continued to be used in basic researches for more than ten years, performing nuclear spectroscopy studies and an important high-precision measurement, with months of data-taking, searching for possible effects of parity mixing in nuclei [1, 2].

But in the mid-1980s, the perspectives for this kind of machines to produce new basic nuclear physics results were becoming weaker and weaker, and also this second life of our Van de Graaff was clearly approaching its end. Another “reconversion” took then place, allowing for a third life of the accelerator through the change of its research goals: from basic to applied Nuclear Physics. Ion Beam Analysis applications (mostly using Particle-Induced X-ray Emission*,* PIXE) were progressively developed at the KN3000. We were among the pioneers in the use of external beam set-ups, e.g. particle beams extracted—through a thin exit window—from the vacuum beamlines of the accelerator, which enormously facilitated all the applications: the use of external beams for the large majority of experiments has since then remained one of the characterizing features of our laboratory, as will be discussed in the following. Already in the first PIXE set-ups, two detectors (at those times, Si(Li)) were simultaneously used to optimize the detection efficiency over a very large X-ray energy range, altogether from ~ 1 up to ~ 30 keV (the low-energy X-rays were effectively detected thanks to the continuous flow of helium in front of one of the two X-ray detectors); thus, all elements from Na to the heaviest were very well detectable through either their K, L or M X-ray lines.

The very first applications we performed dealt with air pollution investigations, for which we developed a home-made sampler for the collection of aerosols with one hour resolution (streaker) and we could obtain a time-resolved compositional analysis of the aerosol collected on the filters [3].

Our attention also focused on applications in cultural heritage problems. At those times, first attempts to use Ion Beam Analysis in the field of cultural heritage had been just started in other laboratories worldwide and we believed that Florence, with its immense tradition and wealth of treasures, was the right place to develop such activities. Although several colleagues of the humanistic disciplines were initially halting and/or concerned about possible risks of damage to the artworks, we eventually succeeded in convincing them that properly operated PIXE measurements might be the right tool to get an answer to some of their questions. With increasing experience and through the development of further dedicated set-ups [4, 5], we could obtain a number of significant results in extensive applications to artworks and documents of great historical importance [6]. In turn, this progressively increased the trust of the humanistic community in the potential and reliability of our techniques, leading to more and more important collaborations.

In parallel, the activity concerning aerosols continued (using the external beam set-up as well) and it was in these applications that we first extensively used also Particle-Induced Gamma-ray Emission (PIGE) technique, complementing PIXE in the analysis of very low-Z elements. Rutherford or Elastic Backscattering Spectrometry (RBS/EBS), with EBS being the general extension to higher beam energies, where the elastic scattering cross section does not follow the Rutherford formula anymore, became routinely employed, too, also with external beams. It was just to address to problem of the lack of cross section data for elastic scattering of protons on low-Z nuclei for the application of EBS, in the absence of theoretically evaluated data, that we started extensive measurements of such cross sections [7–9], that were later included in the IAEA Ion Beam Analysis Nuclear Data Library, IBANDL [10].

Around the turn of the century, with the transfer of the research and teaching activities of Physics to the new University Campus in Sesto Fiorentino, the University of Florence put the budget for a dedicated building to host a larger accelerator and a number of ancillary laboratories. The INFN Executive Board, in turn, decided to fully fund the instrumentation for installing a new applied nuclear physics laboratory. Thus, we were able to buy a 3 MV Tandem, which could expand our IBA potential (through the possibility of higher energies and of a larger range of ions) and start an entirely new activity in Accelerator Mass Spectrometry, AMS, with the goal to establish a laboratory branch for radiocarbon dating (although at that time it was not obvious that a same accelerator might be reliably used for both IBA and AMS).

In April 2003 the dedicated building in the campus was completed; a few days afterwards the new accelerator, a Tandetron built by High Voltage Engineering Europe, was delivered to INFN Florence. Commissioning of sources, injection lines, accelerator, and AMS high-energy beamline was completed one year later in collaboration and with the supervision of HVEE. In the meantime, we had transferred the existing set-ups for IBA from the old accelerator to the new lab, where we also installed further home-designed beamlines and set-ups, including an external scanning ion microprobe. In parallel, the sample preparation laboratory for radiocarbon AMS analysis was designed and installed, and since mid-2004, both AMS and IBA were operational at the Tandetron. The new laboratory, which since then was given the name LABEC, started its activity, and the idea of performing both AMS and IBA at the same accelerator proved to have come true: it was indeed straightforward to switch from one mode of operation to the other with no significant dead times.

Since then, the beamtime allocation between AMS and IBA is roughly equal; for instance, the beamtime distribution in the past five years, excluding maintenance periods, has been roughly: 40% for ^14^C AMS measurements, 40% for IBA measurements (mainly for atmospheric aerosol and cultural heritage studies, but also material science and forensics), and the remaining 20% for irradiation, detector testing and measurements of nuclear cross sections of interest for quantitative IBA applications. Access to LABEC beamtime for external users from public and private institutions and industry is presently possible through the transnational access offered by the European Community H2020 projects RADIATE for IBA measurements (www.ionbeamcenters.eu/radiate/radiate-transnational-access) and IPERION-HS for ^14^C AMS measurements (www.iperionhs.eu/iperion-hsaccess), or through fee-paying third-party services (chnet.infn.it/en/price-list). LABEC is also offering opportunities of knowledge exchange and transfer, and scientific training on accelerator-related analytical techniques to researchers (typically from developing or low-performing countries) through IAEA technical cooperation programmes.

Presently (end of 2020) the full-time LABEC staff consists in nine researchers and four technologists (all physicists, with INFN or University permanent positions at various levels), nine post-docs, with various scientific background (heritage science, physics, chemistry) and two mechanical technicians. Several graduate and master students (three or four, in average), mainly in heritage science and physics, attend yearly LABEC to work on their theses.

Excluding the costs for the permanent personnel salaries, which are in charge of the central INFN administration and of the University of Florence, each for their respective personnel units, the basic annual budget of LABEC for standard operation consumables, instrumentation maintenance, replacements of obsolete or damaged equipment, travel expenses, etc., comes from INFN, directly to its Florence Unit (70 k€ per year) or indirectly through an annual money transfer of 55 k€ to the Department of Physics and Astronomy of the Florence University (mainly used for personnel recruitment—grants and post-doc positions). The University covers the costs related to the building, including energy, cleaning, air conditioning, building maintenance. Additional budget for specific experiments, in the order of several tens of k€ per year as an average, comes from INFN R&D projects; some third-party service also provides some (limited) extra budget. However, increasingly in the past few years, the most consistent amount of money is obtained from competitive projects funded by the Tuscany Region, by the Italian Research Ministry and by the European Community. The amount of this additional budget is variable, depending on the years, but an average value throughout the past five years has been around 300 k€ per year, and has been used—besides of course for implementing the project deliverables—also to cover the costs of the fixed-term positions and grants. Considering European Community funded projects, LABEC is presently a partner of the research infrastructures on ion beam technology applications for fundamental, applied and industrial research with (RADIATE, www.ionbeamcenters.eu), on heritage science (IPERION-HS, http://www.iperionhs.eu), on data for archaeological community (ARIADNEplus, www.ariadne-infrastructure.eu), and on open science and e-infrastructures (EOSC-Pillar, www.eosc-pillar.eu). LABEC has been also a participant to the LIFE + AIRUSE project (www.airuse.eu), which analysed air pollution in five cities in southern Europe, formulating recommendations for effective actions to reduce levels of airborne particles, and earned the People’s Choice 2018 LIFE Award. As regards its involvement in environmental research infrastructures, LABEC is also currently part of the European Centre for Aerosol Calibration, ECAC (www.actris-ecac.eu), of the Aerosols, Clouds and Trace gases Research Infrastructure (ACTRIS), hosting the European reference centre for the elemental characterization of atmospheric aerosols.

## Accelerator and ion sources

**Fig. 1:**
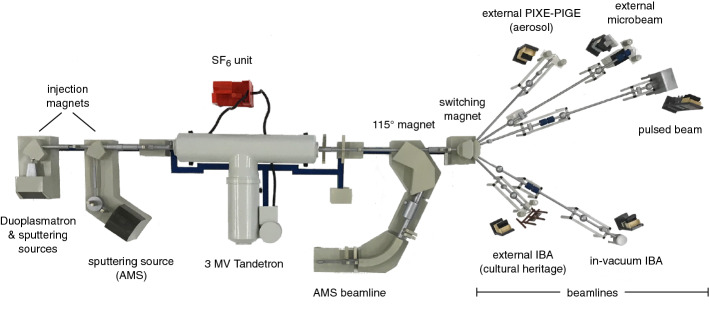
Layout of the INFN LABEC accelerator

Our accelerator is a Tandetron (see Fig. 1) manufactured by High Voltage Engineering Europe, with 3 MV maximum terminal voltage. As is known, Tandetrons are electrostatic Tandem accelerators, where the central terminal voltage is produced by an electronic voltage multiplier [11]. Stripping at the high voltage (HV) terminal is obtained through a light gas flow (Ar), continuously recirculated within the terminal by a locally installed turbo-pump, rotating at half velocity of its standard operating conditions to increase its lifetime. The accelerating tubes, the HV terminal and the charging column are enclosed in a tank filled with SF_6_ gas, at a pressure of 7·10^5^ Pa.

With respect to Tandems where high voltage is produced exploiting continuously moving belts or similar, with Tandetrons tank opening for maintenance is much less frequent. So far, since 2004 when the final acceptance tests of the accelerator have been performed, we had to open the tank only four times.

The flanges of the various parts of the beamlines and associated connections are all ConFlat, with Cu gaskets. The vacuum systems are based on hydrocarbon-free turbomolecular pumps in all sections, backed by scroll pumps. The choice of connecting flanges and pumping systems has been taken to minimize contamination from carbon-containing residual gases, which is very important for radiocarbon concentration measurements.

Three independent sources are available to produce the negative ions to be accelerated towards the positive HV terminal in the first acceleration tube.

Two of them, a Duoplasmatron (HVEE 358) and a single-cathode Cs-sputtering source (HVEE 860), are hosted inside the same cabinet, injecting the produced ions into the subsequent beamlines with an angular difference of 14° between the beam directions from the two sources. The Duoplasmatron source is kept at 20 kV with respect to ground, the Cs-sputtering at − 35 kV. The former is used to produce beams from gaseous elements (different bottles can be located inside the cabinet); the Cs-sputtering source produces negative ions from any solid material.

With the Duoplasmatron, mainly H^−^ ions are produced in order to eventually obtain, at the exit from the Tandetron, accelerated protons. With the exception of He, so far other gaseous elements have only been used very rarely. ^4^He^+^ and ^4^He^++^ (alpha) beams, instead, have been obtained, although not so frequently, after the final acceleration, by feeding the source with He gas. In that case the ions exiting the Duoplasmatron are mainly positive, so the source in this case is kept at + 20 kV, and after the pre-acceleration to 20 keV the positive ions are converted into negative by passing through a Li-vapour charge-exchange canal. The maximum extracted currents from this source are typically several tens of μA for H^−^ ions, and a factor of 10–20 lesser for ^4^He^−^ (after the charge-exchange canal).

With the single-cathode Cs-sputtering source, various kinds of ions have been extracted for experiments carried out during the past few years: most frequently Li (a good alternative to μ particles for standard RBS analysis) and Si beams have been used. This source can also be used to finally produce accelerated proton beams, by using TiH_2_ as the solid material to be sputtered. In this case, the maximum extracted H^−^ current from the sputtering source is lower than with the Duoplasmatron (a few μA).

The ions produced in the two sources are mass-analysed by an injection dipole magnet (83° and 97° deflection, respectively, for ions coming from the Cs-sputtering and the Duoplasmatron) and transported to the accelerator entrance.

The third source, dedicated to AMS measurements, is a multi-cathode Cs-sputtering ion source (HVEE 846b), used so far almost exclusively with solid graphite prepared from samples for ^14^C concentration measurements. While in the sputtering position, samples can be moved in the transversal plane to sputter different positions, so that the possibility to form craters on the graphite surface is minimized. (Otherwise, those craters would affect the electric field inside the source volume.) The extracted particles are analysed according to their mass, charge and energy: an electrostatic analyser is followed by an analysing magnet. The magnet is equipped with a “bouncing” mechanism: the magnetic field is kept constant, but the chamber between the polar expansions is isolated from ground potential and is sequentially biased to different voltage values so that the ion energy during their path in the magnetic field is properly changed in such a way as to yield the same trajectory for the three different masses (14–13–12) to be sequentially injected into the acceleration tube downstream. Injection times for the three masses (8 ms for ^14^C, 600 μs for ^13^C and 6 μs for ^12^C) are set in order not to significantly “lose” ^14^C isotopes, while for the two stable isotopes they keep into account the 13/12 isotopic ratio of about 100, in order to have similar ^12^C and ^13^C counting statistics after acceleration (measuring with accuracy also the 13/12 ratio is important for isotopic fractionation corrections).

Focusing in all the beam transport sections before entering the accelerator is obtained with Einzel lenses, and x and y corrections with electrostatic dipoles. Variable-aperture slits and retractable apertures are also present in these beam transport sections, both before and after the analysing magnets. Monitoring devices such as beam profile monitors and retractable Faraday cups are also present for non-destructive (transmitting) or destructive (intercepting) beam diagnostic.

Thanks to a number of factory-design features of the tubes, the transmission efficiency from the entrance to the exit of the accelerator is high. For protons, we can achieve a transmission efficiency higher than 50%, which means beam intensities that can attain, using the Duoplasmatron source fed with Hydrogen, above 10 μA (although most often in our applications the required current intensities are even many orders of magnitude lower). For heavy ions, the transmission efficiency for the desired final positive charge state (and consequently ion final energy) depends just on the charge state, on the terminal voltage, on the quantity of charge-exchanging Ar gas we flow into the HV terminal. A typical value for C^3+^ ions, using a terminal voltage of 2.5 MV, is around 50%.

We have worked to extend to much lower values the 500–3000 kV range of accelerating voltages for which the system was designed, in particular for implantation experiments for quantum applications, usually performed at an ion energy of few tens of keV since only shallow centres are commonly employed. In these applications it is necessary to greatly extend the original range towards much lower energies, in order to exploit the whole range from a few tens to several thousands of keV and to obtain a variability in implantation depth for the fabrication of devices typically unavailable, especially up to high energies, in the accelerators commonly employed in ion implantation. The minimum employable accelerating voltage is limited to 50 kV by the increased scattering in the stripper and by the out-of-design regime at which the switching magnet and the magnetic steering and focusing system in the beamlines operate. Consequently, the actually available range of the ion energies, exploiting the different charge states of the ion species to be implanted, spans from 135 keV to about 15 MeV, with ion fluences ranging from 10^8^ to 10^14^ cm^−2^ both on large areas (mm^2^) and on small areas, down to the diffraction limit (~ 1 μm^2^, shaping the beam by means of a pin-hole).

## Accelerator beamlines

When not performing AMS measurements, the 115° magnet (see below) installed at about 2 m after the exit of the Tandetron is obviously kept off. As in the low-energy part of the beam transport, x and y electrostatic deflectors, non-destructive (transmitting) and destructive (intercepting) beam monitoring systems are present after the accelerator; standard focusing for all the beamlines after the exit ports of the switching magnet (available at 0°, ± 10°, ± 20°, ± 30°, and ± 45°), is provided before switching by an electrostatic quadrupole triplet just at the accelerator exit and an electrostatic quadrupole doublet before the switching magnet. Depending on the specific kinds of measurements to which they have been dedicated, some of the beamlines, as described in the following, are provided with further beam focusing systems. Currently, the INFN LABEC accelerator has a total of five operational beamlines after the switching magnet (besides the one for AMS measurements), which are used for ion beam analysis and implantation. A sixth beamline is under construction after the + 20° exit port of the switching, at the end of which a vacuum chamber will be installed for large area irradiation of devices with a few MeV energy protons. LABEC is rather unique in comparison to other ion beam laboratories since three of the five beamlines for IBA are dedicated to measurements with beams extracted to ambient pressure. IBA techniques performed while maintaining the target in atmosphere avoids the need of picking up samples, reduces the risk of damage from charge and heat effects and of selective loss of some more volatile elements, greatly ease the object positioning (thus, precious and big artefacts can be studied, for example, for cultural heritage), increase measurement throughput (thus a large number of samples can be analysed in short time—which is important, for example, in studies of long time series of atmospheric particulate matter samples for air quality and climate change issues). Each of the five beamlines will be now briefly described (indicated with the angle of the exit port of the switching magnet), although many share some common features, such as standard beam diagnostic stages (insertable Faraday cup and quartz beam-viewer), or fast-acting UHV valves, mounted in the three lines for external beam measurements just after the switching magnet, to protect the accelerator from air inrush into the beamline, in case of window rupture.

### Beamline at + 45°

The beamline at + 45° is mainly devoted to external beam measurements of cultural heritage. The capabilities of external IBA methods at INFN LABEC laboratory for cultural heritage studies have been improved in the past few years implementing a “Total-IBA” analytical approach [12]. Whereas PIXE, PIGE and EBS/RBS separately give only partial information on the composition and layering of artistic and historical artefacts, these analyses can be performed simultaneously on the sample and their synergistic use allows gathering detailed and complete data about elemental composition and depth distribution of the analysed material. To this purpose the already existing PIXE set-up installed at the + 45° external collimated beam line [13] was upgraded. Moreover, the capability of performing measurements at different beam energies (for instance, spanning from 5 to 2 MeV for protons) in very fast sequences, just recalling pre-set configurations of accelerator terminal voltage and relevant beam transport parameters, allows us to implement efficiently also “Differential PIXE” measurements [14, 15], where a layered, or heterogeneous in depth, material is analysed (probing different thicknesses below the surface at various beam energies).

**Fig. 2:**
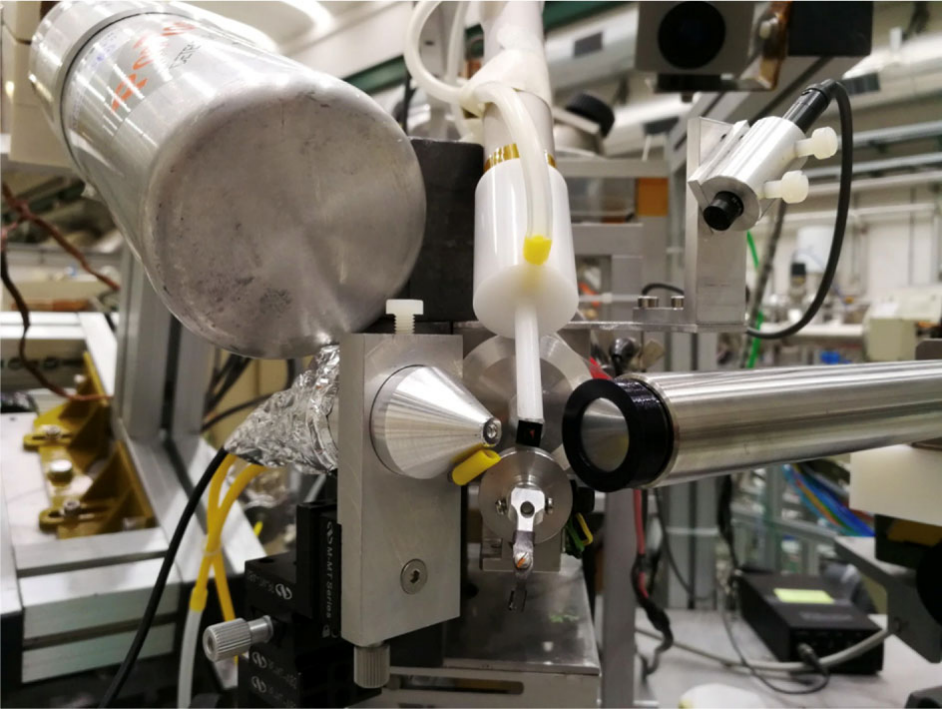
View of the set-up for external beam IBA measurements of cultural heritage on the + 45° beamline at LABEC. Starting from centre top and going clockwise, there can be seen the small area SDD for PIXE with the tube for He flowing, a microcamera for sample inspection from remote, the large area SDD, the rotating chopper, the aluminium case for the EBS/RBS detector and the HPGe detector for PIGE

Here we briefly recall that the beam, typically a proton beam of 3 MeV energy in vacuum and collimated at 0.5 mm diameter, is extracted into ambient pressure through a 200 nm thick Si_3_N_4_ membrane. The sample is placed at about 8 mm from the extraction window, on a *x*–*y* remotely controlled movable stage with micrometric resolution. A laser positioning system is used to properly align and place the sample. A chopper made of Ni evaporated over a graphite vane rotating in front of the target is used to measure the extracted beam weak currents by measuring the Ni *X*-ray yield [16] and to obtain an accurate charge-equivalent normalization for quantitative analysis. Typical used proton beam currents range from a few to several hundreds of pA. A new system to measure the beam intensity, based on counting the Si *X*-rays emitted from the exit window traversed by the beam (see 3.4), is currently under installation and will replace the chopper. The new detector set-up includes (see Fig. 2) now two X-ray detectors for PIXE, a 10 mm^2^ (typically collimated at about 4 mm^2^) Ketek Silicon Drift Detector (SDD), 450 μm thick, with 8 μm Be entrance window, placed 45 mm far from target at 135° angle in the vertical plane, with He flow to reduce the absorption of low-energy *x*-rays, for light and major elements analysis, and a 150 mm^2^ Ketek SDD, 450 μm thick, with 25 μm Be entrance window and an additional 450 μm Mylar absorber to attenuate the low-energy *x*-rays (other absorbers can be inserted if needed), placed 20 mm far from target at 135° angle in the horizontal plane, for heavy and trace elements analysis. A Hamamatsu Si-PIN diode is used as particle detector for EBS/RBS, 10 × 10 mm^2^ active area (collimated to 2.5 × 5 mm^2^), 300 μm thick, placed at 135° scattering angle and mounted 25 mm far from the sample in an aluminium case, kept at 10^–1^ mbar pressure and closed by a 2-μm-thick aluminized Mylar window facing the target. Finally, an ORTEC HPGe gamma-ray detector with a mechanical cooler, 23% relative efficiency, placed at 135° angle, is used for PIGE.

This set-up has been recently used to analyse, amongst others, glazed ceramics [17], coins [18], wall-painting fragments [19] and wall-leather coverings, as well as trace evidences for forensics science applications. In the past, a similar although less complete set-up, using two Si(Li) detectors for PIXE and a planar Ge detector for PIGE, had been used for several important campaigns on even very precious artworks [20].

It has to be noted that the capability of extracting proton beams with extremely low but controlled intensity (currents in the pA range or below), benefitting from the simplicity of sample positioning as given by an external beam set-up, can be exploited also in other applications beside cultural heritage, for example for direct testing of novel detectors and devices with protons of energies up to 6 MeV.

### Beamline at + 30°

The beamline at + 30° is devoted to multi-purpose measurements both for IBA, detector testing and fundamental research under vacuum conditions [21, 22], using a dedicated scattering chamber. The vacuum pressure in the chamber is as low as 1·10^–6^ mbar and pumping is accomplished by means of an hydrocarbon-free system consisting in a dry turbo-molecular pump backed by a scroll fore-pump. A magnetic quadrupole doublet located half-way along the line, 3.5 m upstream of the target, focuses the beam to dimensions of several mm^2^ or smaller on the target.

**Fig. 3:**
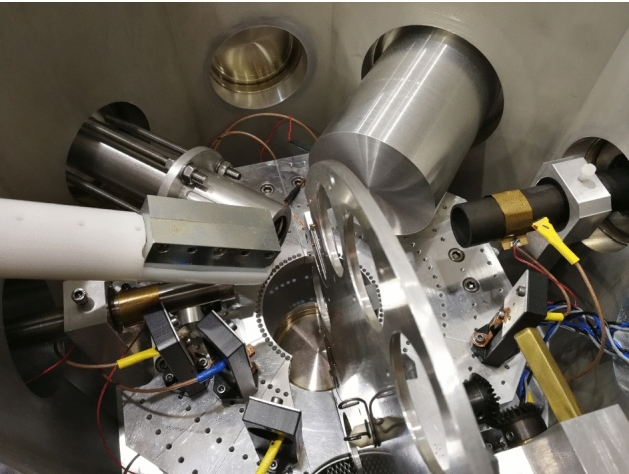
View of the set-up for in vacuum IBA measurements in the scattering chamber on the + 30° beamline at LABEC. Starting from left and going clockwise, there can be seen the small area SDD for PIXE with the proton magnetic deflector, the flange for the insertion of the large area SDD for PIXE, the flange for the insertion of the HPGe detector for PIGE, the Faraday cup, the particle detector for PESA, the sample holder wheel, and the three particle detectors for EBS/RBS

The flexible detection set-up inside the scattering chamber (see Fig. 3) allows for Total-IBA measurements. Elastically scattered ions can be detected simultaneously by three Hamamatsu Si-PIN diodes (10 mm^2^ area, 300 μm thickness) for RBS/EBS measurements. These detectors are placed in IBM geometry at scattering angles of 165°, 150° and 120° and are collimated by 1 × 13 mm^2^ vertical slits set at 61 and 91 mm from the target, for the detector at 150° and for the other two, respectively; apart from this standard geometrical configuration that improves at the same time both mass resolution (with the detector placed a 165°) and depth resolution (with the detector placed a 120°), the particle detectors can be mounted at different angles, from 165° to 110° with 5° steps, at four fixed distances from the target, namely 31, 61, 91 and 121 mm. A fourth similar particle detector is placed at a 30° forward scattering angle, at 121 mm distance from the target and collimated by a 1 × 2 mm^2^ vertical slit, and is used typically for hydrogen detection in aerosol samples collected on thin Teflon filters applying the Particle Elastic Scattering Analysis (PESA) technique [23, 24]. Two X-ray detectors are used for PIXE. A 7 mm^2^ Ketek Silicon Drift Detector (SDD), 300 μm thick, with 8 μm Be entrance window, placed in vacuum 110 mm far from target at 150° angle in the vertical plane, is used for light and major elements analysis. It is equipped with a couple of NdFeB permanent magnets (0.5 T, length 7 cm, distance between magnets 8 mm) in front of it to divert backscattered protons preventing damage to the detector and worsening of the detector energy resolution. A 80 mm^2^ Ketek SDD, 450 μm thick, with 25 μm Be entrance window and an additional 400 μm Mylar absorber to attenuate the low-energy X-rays (other absorbers can be inserted if needed), placed 30 mm far from target at 135° angle in the horizontal plane is used for heavy and trace elements analysis; this detector is inserted in the chamber through an inner protruding open flange, closed at the vacuum side by a 25 μm thick Upilex foil. An HPGe detector is used for PIGE, with a nominal efficiency of 25%, placed at 60 mm from the target at 45° angle in the horizontal plane, inserted in the chamber through an inner protruding Al flange with a 1 mm thick wall facing the target.

The targets are mounted on a sample-holder wheel, that allows the remotely controlled movement of the samples on the x–y axes (perpendicular to beam direction) to scan all the sample surface or to aim at a specific portion of the target, and the change of the samples by rotation of the sample-holder wheel. The beam current, in case of analysis of thin samples, is measured using a long cylindrical graphite Faraday Cup with thick Ta bottom, placed behind the target. In case of thick samples, the current is measured directly from the target. Faraday cup and target can be positively biased up to + 300 V to avoid secondary electron escape.

Apart from IBA, the beamline and the scattering chamber have been used also for the measurements of nuclear cross sections of interest for analytical applications, for instance, for differential cross sections and thick target yields of gamma-ray producing reactions of protons on low-Z targets [25–27], for PIGE applications, installing an additional array of three large volume HPGe detectors at 0°, 45° and 90° angle with respect to the beam direction around the scattering chamber.

### Beamline at − 20°

The 9 m-long beamline placed 20° leftward after the switching magnet is equipped with an electrostatic deflector (DEFEL) system to form pulsed beams down to a few nanoseconds duration [28, 29]. The system is routinely employed both for test of ion detectors and associated front end electronics [30–32] and for ion implantation experiments [33–36]. The latter are designed for the creation of colour centres in solid state matrices, with optical emission properties suitable for quantum applications.

**Fig. 4:**
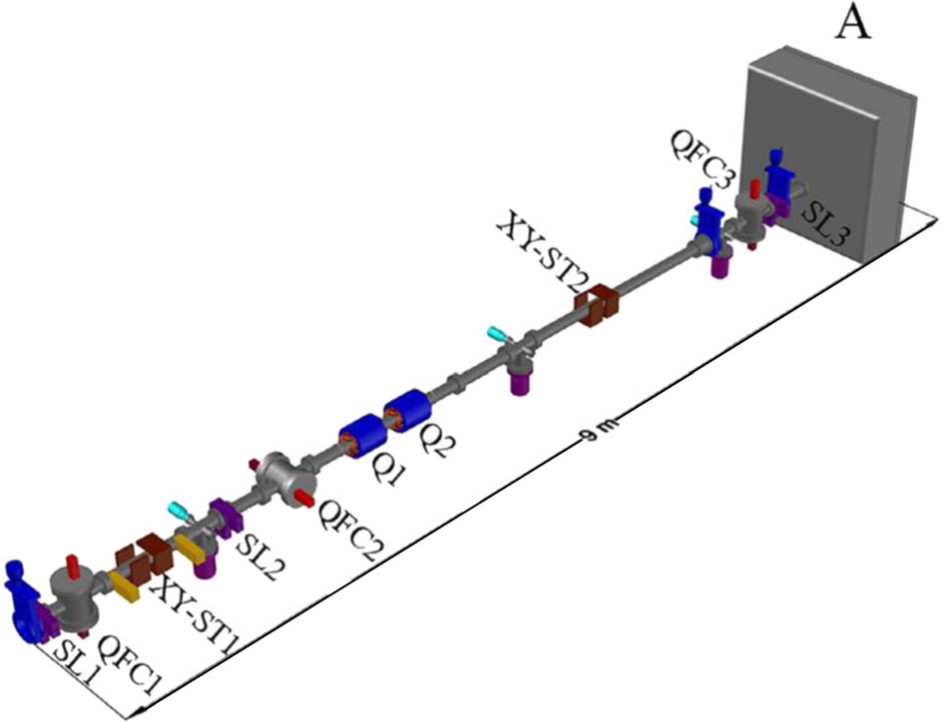
Layout of the DEFEL beamline: SL1-2–3, shaping slits; QFC1-2–3, beam monitor stations allowing insertion of a luminescent quartz plate or a Faraday cup along the beam path; XY-ST1-2, magnetic steerers; A, vacuum chamber. The deflector is installed in the beampipe inside the Q1-Q2 magnetic quadrupoles, the pre-deflector just before it

The electrostatic deflector system is composed of two orthogonally-arranged parallel-plates deflectors: the one closer to the exit slits (SL3), installed in the beampipe inside two magnetic quadrupoles (Q1-Q2, see Fig. 4), is fed with square wave pulses of potential difference from + 100 V to − 100 V, creating a very short (a few ns) bunch passing through the exit slits. Before this deflector, an 800 V–0 V square wave pulse is applied to a pre-deflector, so that only the falling part of the deflector square wave pulse, having a different duration compared with the rising part, can send the beam to the exit slits. In a different configuration, only the pre-deflector is activated, to form beams 1–5 μs long if requested. The DEFEL beam line is also equipped with two magnetic quadrupoles (Q1–Q2) focalizing the object formed by an entrance slits (SL1) on the plane of the exit slits (SL3), with a nearly unitary magnification factor. The divergence of the beam is also limited by another pair of slits (SL2) before the quadrupoles, and the beam direction is controlled by two pairs of steering magnets (XY-ST1-2). Beam monitoring is performed by three standard beam diagnostic stages placed along the line (QFC1-2–3), each with a Faraday cup and a quartz beam-viewer.

The full pre-deflector + deflector system is mainly employed for detector testing since it can reach, with its very short pulse width, the Poisson statistic “one particle-per-bunch” regime on areas of the order of 1 mm^2^–1 cm^2^. On the other hand, the ~ 1 μs pulses obtained with the pre-deflector alone can be used to reach the “one particle-per-bunch” regime across beam-shapers of micrometric diameters, which are especially employed in ion implantation experiment designed to place single-photon sources in solid state samples with spatial resolution comparable to the diffraction limit (a few hundreds of nm). The same line, with the deflectors off, is employed to implant at high fluences (10^12^–10^15^ cm^−2^) and to obtain luminescent properties of the implanted centres detectable at ensemble level.

At the end of the beamline a half-m^3^ vacuum chamber, allowing a great versatility in testing-implantation geometries, is installed. During the implantation experiments, in particular, a variety of monitoring devices is employed: a Faraday cup and a PIN diode to monitor the beam current in continuum-pulsed regime, a camera to aim a perforated quartz behind which the micrometric beam-shapers are placed, a microscope equipped with a high sensitivity camera observing the luminescence produced by the shaped beam on a transparent sapphire window, in order to aim the sample which is moved behind the beam shaper by two motorized precision linear stages with nanometric nominal resolution.

### Beamline at − 30°

The external ion microprobe of the INFN-LABEC laboratory is installed on the -30° beamline [37, 38]. The optical elements before the switching magnet (electrostatic quadrupole triplet and doublet) produce a beam waist about 2 m after the switching magnet; in correspondence of the beam waist, the object slits of the microbeam are installed. Vertical and horizontal steering magnets, just after the switching magnet, allows the alignment of the beam along the beam optical axis. Downstream the remotely controlled object slits, a beam diagnostic stage including a beam profile monitor (BPM) is installed. About 6 m after the object slits, a second pair of steering magnets allows aligning the beam along the magnetic axis of the subsequent system, composed of the beam scanning coils and a magnetic quadrupole doublet (by Oxford Microbeams Ltd.). Collimation slits, a second BPM and the quadrupole doublet are mounted on common granite base plate. About 20 cm downstream of the second quadrupole, the beam is extracted in atmosphere, either air or He, passing through the exit window, typically a 100 nm thick Si_3_N_4_ membrane, and hit the target, at a typical distance of 2–3 mm.

A SDD, positioned under the nozzle, at about 40° with respect to the beam direction, counts the Si X-rays emitted from the exit window traversed by the beam. A fraction of these X-rays passes through an aperture in the nozzle rear-side, sealed with a 7.5 μm Upilex foil (50% transmission factor for Si X-rays). The beam impact point on the sample to be analysed, which is placed after the exit window, remains external to the solid angle subtended by this SDD, so that just the Si X-rays produced in the exit window are detected. As the number of the X-rays detected by this detector is proportional to the number of incoming particles, this system allows us to obtain an indirect measurement of the number of particles hitting the sample for quantitative analysis.

The scanning system makes the collection of elemental maps possible, using both the sample displacement under fixed beam and the beam raster-scan over the static sample.

A C +  + control program [39] has been developed together with the control program of our XRF scanner [40]—see below, so they present many common solutions, starting from the user interface. Data acquisition is based on a combined NIM-VME system; the VME part is dedicated to signal digitizing and data writing, the NIM part is used to create the gate signal for the peak-sensing ADC (CAEN V1785). Two motorized precision linear stages allow moving the sample under fixed beam. The sample is in atmosphere in front of the beam and is moved on a horizontal (or vertical) line with constant velocity (set by the user, maximum 10 mm/s). When at the end of the line, the y position (*x*) is incremented by a step (set by the user) and the motion along the x direction reversed. The step is usually set of the order of the beam spot size on sample, normally in between 10 and 30 μm. The spatial resolution is limited by the beam size on sample, being the overall position uncertainty due to the stages (about 6 μm) by far less influent. Otherwise, the use of the scanning coils allows us to move the beam on the static sample on an area as wide as the beam extraction window, up to 2 × 2 mm^2^.

At our laboratory, also heavy-ion high-spatial-resolution probes have been produced [39]. Carbon microbeams with energies in the 10–15 MeV range have been produced using the Cs-sputtering source. Carbon microbeams were extracted in He atmosphere through a 50 nm thick Si_3_N_4_ window, with a 2 mm window-to-target distance. With a 10 MeV carbon microbeam (charge state 4+, terminal voltage 2 MV), we can obtain good intensity, about 1 nA electrical current and size of about 30 μm on sample. Carbon beams with these characteristics, the range of which is a few microns in diamond, could be an interesting tool for the community involved in the fabrication of optical devices in diamond.

**Fig. 5:**
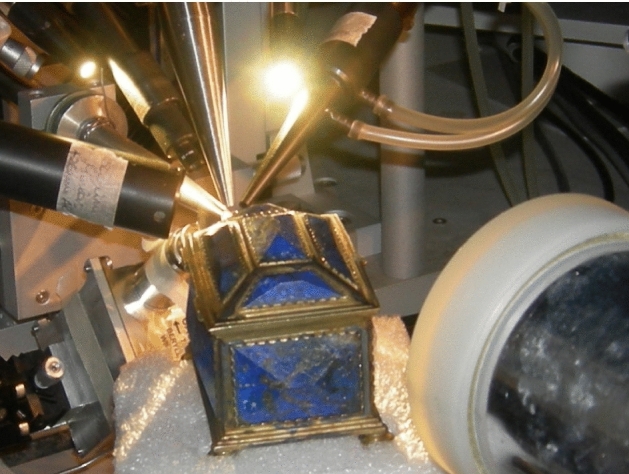
View of the external microbeam set-up during a measurement on one of the lapis lazuli jewels of the Collezione Medicea. In the picture, the PIXE, PIGE and EBS detectors are visible. In the lower part of the photograph, under the beam extraction nozzle, the detector for beam intensity monitoring can also be noted

The detection set-up (see Fig. 5) allows Total-IBA measurements with PIXE, PIGE, EBS and IBIL (Ion Beam Induced Luminescence) techniques. Also, an out-of-vacuum set-up for PESA or Forward Scattering (FS) spectrometry has been developed at the external microbeam [41]. The system is based on two Hamamatsu windowless photodiode particle detectors, 300 μm thick, mounted in vacuum-tight cases sealed with a 2-μm-thick aluminized Mylar, similar to the EBS detector installed in the set-up of the + 45° beamline, mounted over a semi-circular rail, the centre of which is in the point of interaction of the beam on the sample surface. The angle of each detector can be remotely controlled with a 1/40° angular accuracy. Exploiting this set-up, it is possible to perform Scanning Transmission Ion Microscopy (STIM) measurements in the on-axis, off-axis and on–off-axis configurations (these latter two configurations make it possible to perform simultaneous STIM and IBA measurements), using different ions, energies, and scattering geometries.

The Florence external microbeam has been widely used for applications in the cultural heritage field [13, 42, 43], and for measurements of interest in earth sciences [44], material science [45, 46] and biology [47].

### Beamline at − 45°

The beamline at − 45° is devoted to external beam measurements of atmospheric particulate matter samples. For more than 30 years an external beam line fully dedicated to the analysis of aerosol samples has been operational at INFN Florence, and has been constantly improved over the years [48–50] benefitting from the progress in the state-of-the-art technology for X-ray detectors. The present configuration makes the external beam PIXE-PIGE set-up at LABEC the most advanced worldwide for high-throughput analysis of daily particulate matter samples collected on filters, as well as of size-segregated and high-time-resolution aerosol samples.

**Fig. 6:**
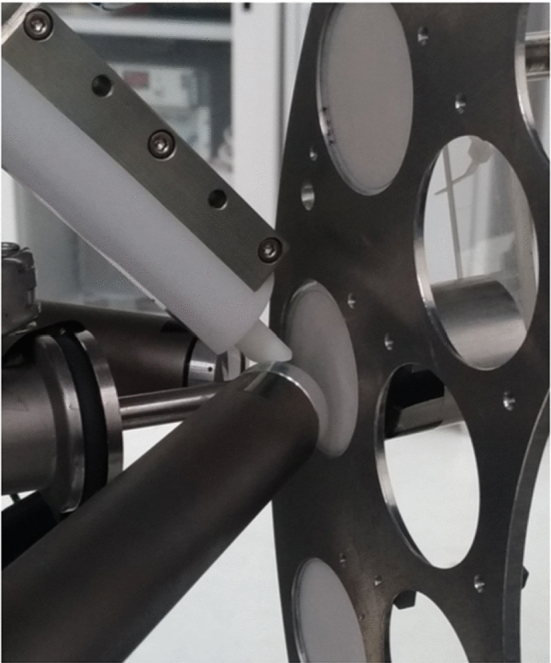
View of the set-up for external beam PIXE-PIGE measurements of atmospheric aerosol samples on the − 45° beamline at LABEC, with the SDD for low-energy X-ray detection (upper part of the picture, showing also the magnetic proton deflector assembly), the twin SDDs for mid- high energy X-ray detection (on the left and the right of the beam extraction nozzle), the filter holder wheel (with daily aerosol samples) and the Faraday cup behind it. The PIGE detector is not shown in this picture

Here the beam is typically a proton beam of energy between 3.2 MeV and 2.7 MeV in vacuum. The energy to be used for a given set of measurements is chosen in such a way as to reduce the background in PIXE spectra according to composition and thickness of the filter used to collect the aerosol (for instance, Teflon, Nuclepore, Polypropylene or Quartz fibre). The beam is extracted into ambient pressure through a 500 nm thick Si_3_N_4_ membrane. The filters with the aerosol deposit are positioned at about 1 cm from this exit window. The beam size is usually 1 × 2 mm^2^, as defined by bare collimation in vacuum in the very last section of the beamline. The beam charge flown during the measurement is measured by simply integrating the beam current on a graphite Faraday cup positioned just behind the (thin) sample. Typical used proton beam currents range from tens to a few hundreds of nA. During irradiations, the samples are continuously moved on the x–y axes (perpendicular to beam direction) in order to scan all the sample surface and to reduce the beam charge density on the sample. This scan is remotely controlled by the acquisition system, which also provides the change of the samples by rotation of the sample holder. The detector set-up (see Fig. 6) includes a 30 mm^2^ Ketek SDD dedicated to low-Z elements, 450 μm thick, with 8 μm Be entrance window, placed at about 90 mm far from target at 135° angle in the vertical plane, with He flow enabling detection of X-rays down to 1 keV, thus the detection of elements down to Na. A couple of NdFeB permanent magnets (0.5 T, length 8 cm, distance between magnets 8 mm) is installed along the path of X-rays from target to detector entrance window to divert backscattered protons, thus avoiding that they reach the detector (producing too large pulses that would saturate the electronics and worsen the detector energy resolution, and possibly damaging to the detector itself). Two identical KETEK 80 mm^2^ SDDs dedicated to medium–high Z elements, 450 μm thick, with 25 μm Be entrance window and 450 μm Mylar absorber to attenuate the low-energy X-rays, placed at about 20–25 mm far from target at 135° angle in the horizontal plane on opposite sides with respect to the beam directions, are used to double the statistics by summing offline their acquired spectra. As a whole, this SDD array covers a total solid angle of 400 msr. For an accurate quantification of elements like Na (key element for the study of marine aerosol), Mg, Al, Si (fundamentals for the study of mineral dust), when the X-ray attenuation inside the sample cannot be neglected and cannot be calculated either considering a homogeneous average composition of the aerosol sample (because aerosol samples contain particles of different composition and size with a distribution which is not known ‘‘a priori”) [51–53], PIGE measurements can be performed simultaneously by using a Canberra planar Ge detector of 20% relative efficiency, that can be placed at 45° angle in the forward direction.

### AMS beamline

At LABEC, ^14^C-AMS measurements are routinely performed at 2.5 MV terminal voltage, analysing the 3+ charge state on the high energy side [54]. Both ^14^C/^12^C and ^13^C/^12^C isotopic ratios are measured along the beamline in the same measurement run. After the accelerator, the beam is firstly analysed through a 115° magnet, which is set to transmit 3+ ^14^C ions of 10.035 MeV (the total energy gained from the ion source + acceleration in the Tandem when the terminal voltage, TV, is at 2.5 MV and the charge exchange from 1- to 3+ has taken place just within the TV), while ^12^C and ^13^C of the same charge state and energy are measured through off-trajectory Faraday cups. Downstream the magnet, the beam is further analysed through an electrostatic analyser to suppress possible residual interferences given by non-^14^C that may have undergone charge–exchange interactions along the high-energy accelerator tube, and would have the same energy-mass–charge state combination of the ^14^C 3+ ions of 10.035 MeV. The final part of the beam line has been modified many times, according to the experimental needs. For instance, a Time-of-Flight (TOF) system has been set up [55]. This system was based on the exploitation of secondary electrons emitted by the incoming beam impinging on a thin foil, later amplified thanks to a microchannel plate, which provides a very quick start signal. In our set-up, the stop signal was then given by the same mechanism or, as an alternative, by the Si photodiode itself used for ^14^C counting. The count rate on this Si photodiode is very low (few tens of Hz maximum). The final part of the beam line has been recently upgraded by also installing a position sensitive silicon detector used to monitor the position of the beam with respect to the centre of the line, in the transversal plane [56].

## Quality assurance procedures

The knowledge of the absolute ion beam energy is essential for depth profiling and quantification using particle scattering techniques such as EBS/RBS. It is also important for PIGE since the gamma-ray producing cross sections are sensitive to the particle absolute energy as well. The accelerator terminal voltage energy is measured by a generating voltmeter (GVM). A procedure for precisely determining the absolute energy using known sharp and isolated resonances in nuclear reactions is employed, using (a) a thick aluminium target and the resonances at 991.86 keV and 1683.57 keV, respectively, in ^27^Al(p, γ)^28^Si, E_γ_ = 1779 keV, and ^27^Al (p,p’γ)^27^Al, E_γ_ = 843 keV, reactions; b) a pressed ZnS pellet and the resonance at 3379 keV in ^32^S(p,p’γ)^32^S, E_γ_ = 2230 keV, reaction. A fit to the three calibration points is then performed using a linear relation between the proton energy (*E*) and the nominally set terminal voltage (*TV*), $E=2\cdot \left(a\cdot TV+b\right)+{E}_{source}$, with *E*_*source*_ being the H^−^ energy once extracted from the source (for instance, 20 keV when the Duoplasmatron source is used); the calibration allows the determination of the terminal voltage of the accelerator with an accuracy better than 0.1%. The accelerator energy calibration is typically performed at least once per year.

For IBA quantitative measurements, reference spectra from standard materials are recorded for each measurement run, depending on the sample to be analysed (for instance, thick or thin) and to the employed IBA technique, as listed in Table 1.

**Table 1:** List of standard materials used for obtaining reference spectra for IBA measurements

Technique	Standards	Notes
PIXE	NIST 1412, 612, 620; BCR126; high-purity metal foils	Thick; NIST and BCR are multielemental glass standards
	NIST 2783, MicroMatter XRF standards	Thin; NIST is the atmospheric particulate matter reference sample; MicroMatter standards are mono- or bi-elemental (thicknesses ranging from 15 to 120 μg/cm^2^) deposited on 6.3 μm Mylar
PIGE	NIST 1412, 620; BCR126	Thick, glass standards (for Na, B, Li)
	^132^Ba, ^152^Eu, ^222^Rn	Radioactive calibration sources
RBS/EBS	IRMM-302	Sb implanted in Si/SiO_2_
	High-purity metal foils; BCR126	Thick; BCR is a multielemental glass standard
	MicroMatter XRF standards	Thin; mono- or bi-elemental standards with thicknesses ranging from 15 to 120 μg/cm^2^, deposited on 6.3 μm Mylar

Since LABEC employs many particle, X-ray (SDD) and gamma-ray (HPGe or Ge) detectors, the performance of such devices, in terms of detector energy resolution and energy calibration parameters, are recorded for each measurement run, because they are integral to obtaining accurate analysis of samples and in order to anticipate the need for detector replacement.

For AMS (^14^C measurements) the measured isotopic ratios are typically normalized to Oxalic Acid II (NIST SRM 4990C) primary standard. The evaluation of the “background counts” due to contaminations in the chemical pre-treatments and to machine measurements is performed using nominally ^14^C-free materials. A consistency of the internal accuracy is routinely performed at each beam time with samples prepared from another standard reference material IAEA C7 (secondary standard), and similar control checks but using samples prepared from other standard reference materials such as IAEA C2 (travertine), C4 or C5 (sub fossil wood samples) are periodically performed. Moreover, when the sample mass is sufficient, two graphite fractions are prepared from each pre-treated sample and measured independently, as a more robust check on the presence of contaminants.

## Instrumentation for in situ cultural heritage analysis

Our laboratory is also equipped with a few portable Macro-X-Ray Fluorescence (MA-XRF) scanners specifically customized for heritage science applications, designed and developed in the framework of the CHNet collaboration of the INFN (http://chnet.infn.it). The design of the equipment is primarily focused on portability, for truly compact lightweight system well suited for in situ campaigns [57]. A detailed description, including technical characteristics, analytical capabilities, software tools for data acquisition and elaboration, is given in [40].

**Fig. 7:**
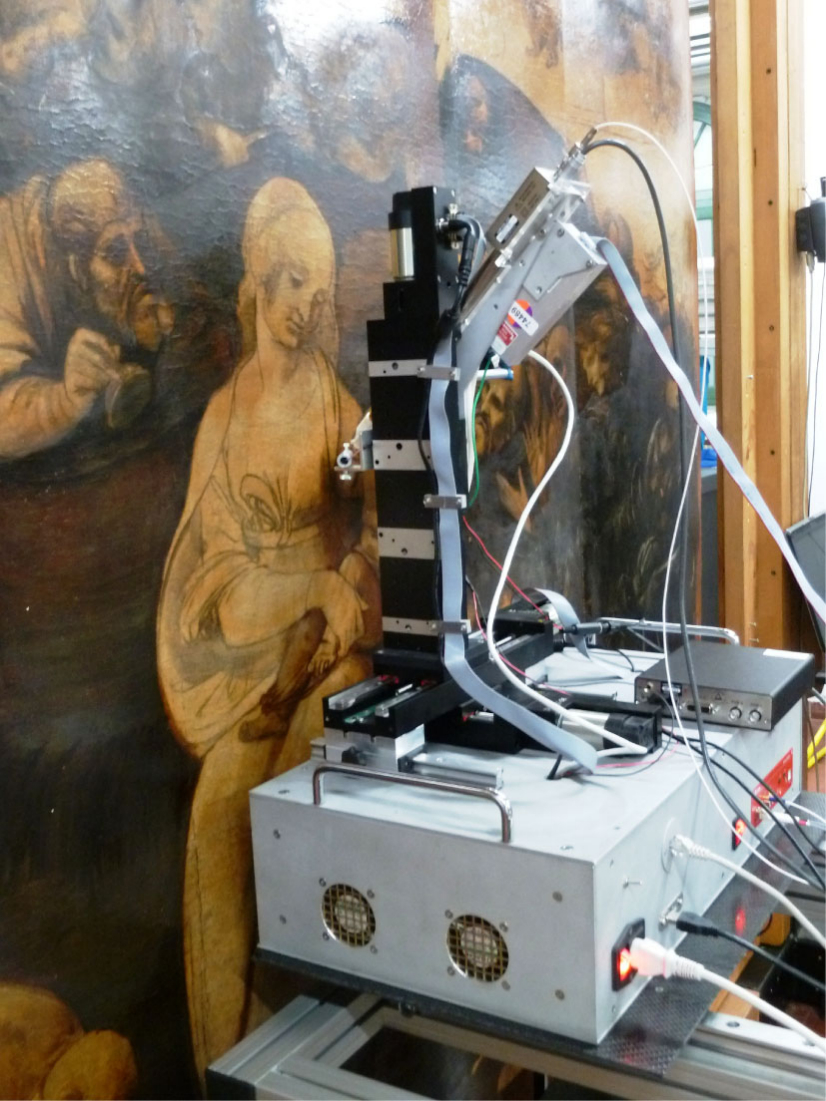
One of the LABEC MA-XRF scanners during the analysis on the Adoration of the Magi by Leonardo da Vinci

Concisely, as shown in Fig. 7, the instrument consists of a measuring head with an X-Ray tube (Moxtek, 40 kV maximum voltage, 0.1 mA maximum anode current, typically Mo anode—tubes with other anode materials are also available), an Amptek SDD (50 mm^2^ area, 500 μm thick, 8 μm Be entrance window) and a telemeter (Keyence IA-100) that continuously measures the sample-instrument distance during the scan, providing a feedback to keep it constant. The measuring head is mounted on three linear motor stages (for the present version 300 mm and 150 mm travel range in the horizontal and vertical directions, respectively, to allow the scan on a plane parallel to the surface to be scanned—plus a 50 mm stage along the “z” perpendicular direction, to actively maintain the correct head-to-sample geometry, thanks to the feedback from the telemeter). This is very helpful when scanning on uneven surfaces such as may be those of panel paintings (nominally flat, but often in practice uneven over large areas) and even more those of manuscript pages; or on truly three-dimensional objects (even over small areas) such as, for example, potteries. The motors are fixed on a carbon-fibre box which contains motor controllers, the detector signal digitizer (CAEN DT5780), cooling fans, power supplies and other auxiliary elements. Supports, holders and other mechanical parts were produced with 3D-printing technology, providing the creative technical ability to build some variants to fully customize the instrument for the different applications encountered. Motion, acquisition and data elaboration are controlled via a software developed by CHNet making use only of open-source programming software (QT platform), which allows us total independence in making changes, improvements and new implementations. Software protocols are fully described in the above-cited reference [40].

The whole scanner weighs less than 10 kg and measures 60 × 50 × 50 cm^3^, so that it can be handled by a person alone, packed in two medium-size boxes. Compared with other instruments with similar performance, it is thus lighter and smaller. The system can also be battery-powered, a feature that comes decisive in campaigns such as those in archaeological sites.

Radiation protection prescripts are respected by delimitating an appropriate no-access area around the instrument by photoelectric sensors that switch off the tube if the area is not free. The X-ray tube has an additional brass shielding to cut residual transmitted radiation.

Some of these spectrometers have been successfully used for a number of cultural heritage applications, ranging from canvas, panel and mural paintings, archaeological finds, coins and metals, porcelains and other manufacts, also thanks to the numerous national and international collaborations as those with the Opificio delle Pietre Dure in Florence and several research groups. In these collaborations, the analyses have been carried out following a multi-technique approach for a comprehensive material characterization, see, for example, [18, 19, 58–61].

In general, however, IBA is much more effective than XRF for the compositional analysis of materials in the field of cultural heritage. This is due to some intrinsic limitations when using XRF in this field (much poorer sensitivity to light elements; difficulty to discriminate the layer structure of the artworks—typically present, for example, in paintings; higher penetration of the primary X-rays with respect to protons, resulting, for example, in the “confusing” presence of contributions from the preparation layer below the paintings). But more than that, the real plus of IBA vs XRF lies in the fact that when doing an irradiation with ions you can simultaneously exploit many different interaction products (X-rays, gamma rays, backscattered particles, etc.) and not just the secondary X-rays obtained using XRF. In other terms, Total-IBA is a “multi-messenger” suite of techniques, each providing—in a single run—information that complements or reinforces the one obtained from the other simultaneously exploited techniques.

**Fig. 8:**
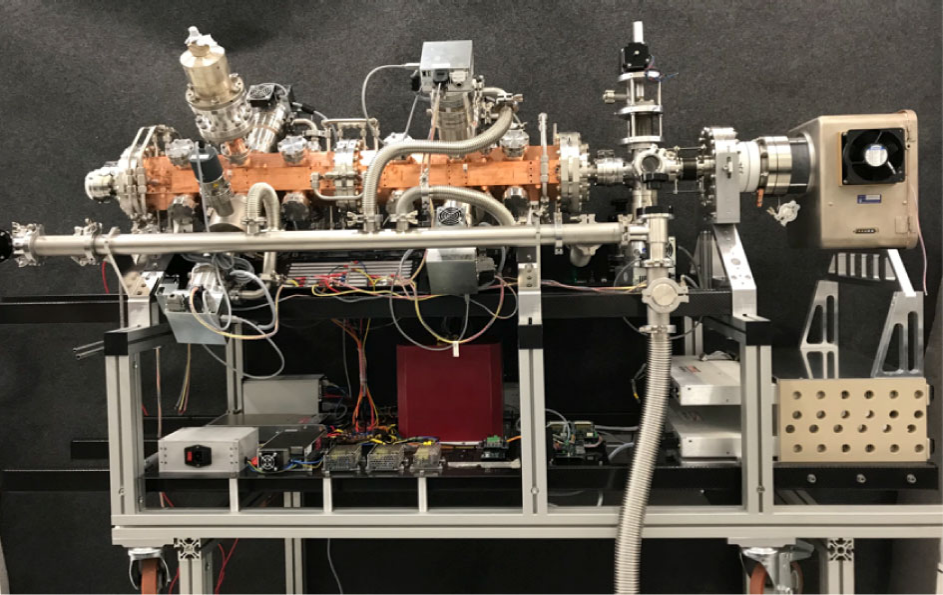
The MACHINA compact accelerator under assembly at LABEC

The only real limitation of IBA remains the need to take the artworks to the accelerator laboratory. Therefore, with the goal of building a transportable instrumentation to be used for IBA also outside an accelerator laboratory, a project (MACHINA, Movable Accelerator for Cultural Heritage In situ Non-destructive Analysis) has been launched at LABEC as a joint initiative with the technical division of CERN. At CERN, relying on their huge experience in accelerator technologies, a dedicated, very compact RFQ (Radio-Frequency Quadrupole) proton accelerator (2 MeV proton energy, 1 m long) [62] has been built for this specific purpose. At LABEC, we have designed and built the pre-acceleration and the high-energy beamlines, the final external beam PIXE-PIGE-EBS set-up, and developed the driving control electronics and software for the whole apparatus, as well as for the vacuum systems. The pre-acceleration beamline contains what is just “essential” and can thus be very compact: after a directly coupled RF source injecting 20 keV protons, it includes anyway all the necessary active beam control and monitoring elements; in the post-acceleration line, also very short, besides magnetic beam focusing and steering elements, insertable energy degraders (to also allow for differential PIXE) are present just before the final external beam set-up. Altogether, the length of the instrument (see Fig. 8) is less than 2.5 m and the weight is around 200 kg.

MACHINA is intended to be first installed at the Opificio delle Pietre Dure, in Florence, one of the most prestigious public Institutions for restoration of artworks in the world, but it is foreseen that the system, thanks to its transportability [63], can be moved to other restoration laboratories or museums for specific diagnostic campaigns.

The project has been presented on several occasions in workshops and conferences, and the whole apparatus will be described in detail in a forthcoming paper.

The completion and commissioning of the system were initially foreseen at the end of 2020, but owing to the severe restrictions to laboratory activities imposed by the COVID-19 pandemic both in Italy and in Switzerland, we are experiencing a delay of about one year. Anyway, the assembled system is by now (end of 2020) basically ready for the final technical commissioning. Immediately afterwards, some months of further checks on test samples are foreseen at LABEC, before starting its actual operation on artworks at OPD, what we still hope to be able to do within one year.

## Aerosol laboratory

The study of atmospheric aerosol is a very multi-disciplinary research field; therefore, besides collaboration with groups with other expertise (e.g. chemists for the determination of the ionic component or other specific compounds, geologists for a deep understanding of crustal dust, etc.), ancillary laboratories are mandatory for optimizing the synergy among aerosol sampling, nuclear analytical techniques and complementary analyses.

Focusing on nuclear techniques, beyond IBA, at LABEC carbonaceous aerosol may be analysed for their radiocarbon content (in separated fractions, namely organic and elemental carbon) by AMS thanks to the custom sample preparation line developed and realized for such purpose. These analyses are unique for the assessment of the contribution to the carbonaceous aerosol load from the different sources, such as biomass burning, fossil fuel combustion and biogenic. A full description of the dedicated sample preparation line may be found in [64, 65].

The LABEC laboratory is equipped with several aerosol sequential samplers for “standard” aerosol sampling (daily resolution on PM10/PM2.5/PM1 fractions according to the European Reference Methods EN12341:2014), and other commercial samplers for the collection of size-segregated samples: namely, DEKATI SDI impactor for the collection of aerosol in 12-dimensional classes [52] and PIXE Int. streaker samplers for the collection of high-time-resolution coarse and fine samples [49]. Furthermore, since commercial samplers, and especially the streaker, not always fulfil the requirements for the sampling campaigns and/or are not any longer available on the market, the group has developed a novel sampler for high-time-resolution and size-resolved sample collection (STRAS—Size- and Time-Resolved Aerosol Sampler).

The laboratory is also equipped with additional instrumentation for complementary analyses. An energy dispersive XRF spectrometer with three-dimensional, polarizing optical geometry and a set of secondary anodes, with automatic sample loading (Epsilon 5 by PANalytical B.V.), is used for routine analyses or coupled to PIXE in order to optimize the beam time request [66]. An organic carbon/elemental carbon (OC/EC) analyser by Sunset Inc. is also available [67]: as IBA measurements do not give information on the carbonaceous content of aerosol (or they only allow it on a few aerosol collection substrata, as reported in [24]), this kind of analyses are very important to obtain a major piece of information (carbonaceous aerosol may account for up to 50% of the aerosol mass in urban environments), complementary to the one provided by IBA in order to be able to completely reconstruct the aerosol mass (mass closure). Besides the EC/OC lab analyser, at LABEC a semi-continuous OC/EC field analyser (by Sunset Inc.) is also available for high-time-resolution campaigns, to be coupled to streaker/STRAS samplings (e.g. [68]).

Beyond instrumentation, aerosol research at LABEC benefits from a deep expertise in the use of statistical tools for aerosol source apportionment (such as Positive Matrix Factorization—PMF analysis) [69].

## Radiocarbon sample preparation laboratory

The sample preparation laboratory for radiocarbon measurements is fully equipped to treat materials like charcoals, wood, seeds, textiles, bones and carbonates [70]. As for the chemical pre-treatment of organic samples, we usually follow the so-called ABA procedure, which is applied when the possible contaminations are only expected to come from the natural environment. In this procedure, samples alternatively undergo baths in acidic and/or basic aqueous solutions to get rid of exogenous carbonates and humic substances. The procedure, e.g. the duration of each preparation step and the molarity of the solutions, is of course adapted according to the material to be treated and to the state of preservation of the samples. Other procedures to be applied when anthropogenic contaminations due to past restorations are expected have been set up and used (see, for instance, the process based on CHCl_3_ to remove resins such as Paraloid B72) [71].

Extraction of carbon from the cleaned material is achieved using a CHN elemental analyser (Thermo Flash EA 1112) and collecting only the CO_2_ evolving from the sample combustion. Carbon dioxide is then converted to solid carbon, i.e. graphite, by reaction of CO_2_ with H_2_, in the presence of Fe as catalyst and at a temperature of about 600 °C. The graphitization line is provided with eight reaction chambers in order to allow for several reactions in parallel, thus improving the sample preparation throughput of the laboratory. Two of the reactors have been recently upgraded to be optimized for the graphitization of samples as small as 50 mg of carbon (as a comparison, the mass of our “traditional” large samples is about 700 mg of carbon) [72]. In these reactors, the internal size is minimized by reducing dead volumes, such as those of the pressure gauge, used to monitor the behaviour and the progress of the reaction, or of the cold finger, which traps the water forming during the graphitization reaction, or of the valve, which isolates the reactor from the rest of the line.

The graphitization line is also equipped with a different gas inlet to allow transferring carbon dioxide evolved from a “device” different from the elemental analyser. For example, in the case of measurements on carbonates, when we would like to only collect specific CO_2_ fractions, CO_2_ is obtained by dissolution in H_3_PO_4_, and is injected into the reaction tube through a syringe. The reaction tube and a water trap downstream are then directly interfaced to the graphitization line [73].

## Success stories

During the over 30 years of activities of our laboratory, besides a lot of more or less routine—although very useful—measurements both in the field of aerosol composition detection and in the one of cultural heritage issues, which were performed to provide always important but “standard” information to the specific stakeholders, we obtained several times also results that for their specific interest or for the importance of the case under study, also gained a wide echo in a wider community. We can quote and we will describe in detail in the following, as to IBA measurements, the cases of elemental analysis of high-time-resolution aerosol sample and, as to AMS measurements, the case of ^14^C measurements on microsamples.

### Elemental analysis of high-time-resolution aerosol samples

In environmental sciences, PIXE (sometimes complemented by other IBA techniques), thanks to its high sensitivity for detecting trace elements, plays an important role through the measurement of the elemental composition of the aerosol. The analysis of samples with very low mass such as those collected with high time resolution is thus possible. In most of the field campaigns, the aerosol is collected with a 24-h time resolution. However, many particulate emissions change within a few hours (industrial or traffic emissions, construction works, etc.); moreover, as many meteorological parameters, like wind intensity and direction, change within a 1-h time scale and the boundary layer evolution shows strong diurnal patterns, atmospheric transport and dilution processes change within a few hours. Consequently, the aerosol concentration and composition may significantly change within a short time and daily samples are not capable of tracking these rapid changes. For this reason, the measurement of the aerosol composition with high time resolution is important to assess health and environmental effects, understand transport processes and determine source contributions. To fully exploit the potential of PIXE in the analysis of aerosol samples, a proper experimental set-up such as the one available at LABEC and described in Sect. 3.5 is important.

Source apportionment analysis by PMF of hourly data reinforces the source identification obtained by daily samples, since the impact of many sources like industries, biomass burning or vehicular traffic is more evident on an hourly time basis. Furthermore, a more direct correlation with wind direction and speed is possible, since on a daily scale the wind direction may have strong variations. Source polar plots, which show the wind direction and intensity dependence of the resolved factors, can be easily produced from PMF streaker results. For example, in the AIRUSE project [74] the time series of the biomass burning source is characterized by a periodic pattern with peaks starting in the evening and lasting several hours, supporting the identification of this source as biomass burning for domestic heating. The absence of the evening-night peak on some days is explained by the meteorological conditions. Some peaks during the day are connected to open fires due to the pruning. Other examples of application are studies in Madrid [75], Barcelona [76], Japan coastal areas [77] or a megalopolis as Beijing [78].

**Fig. 9:**
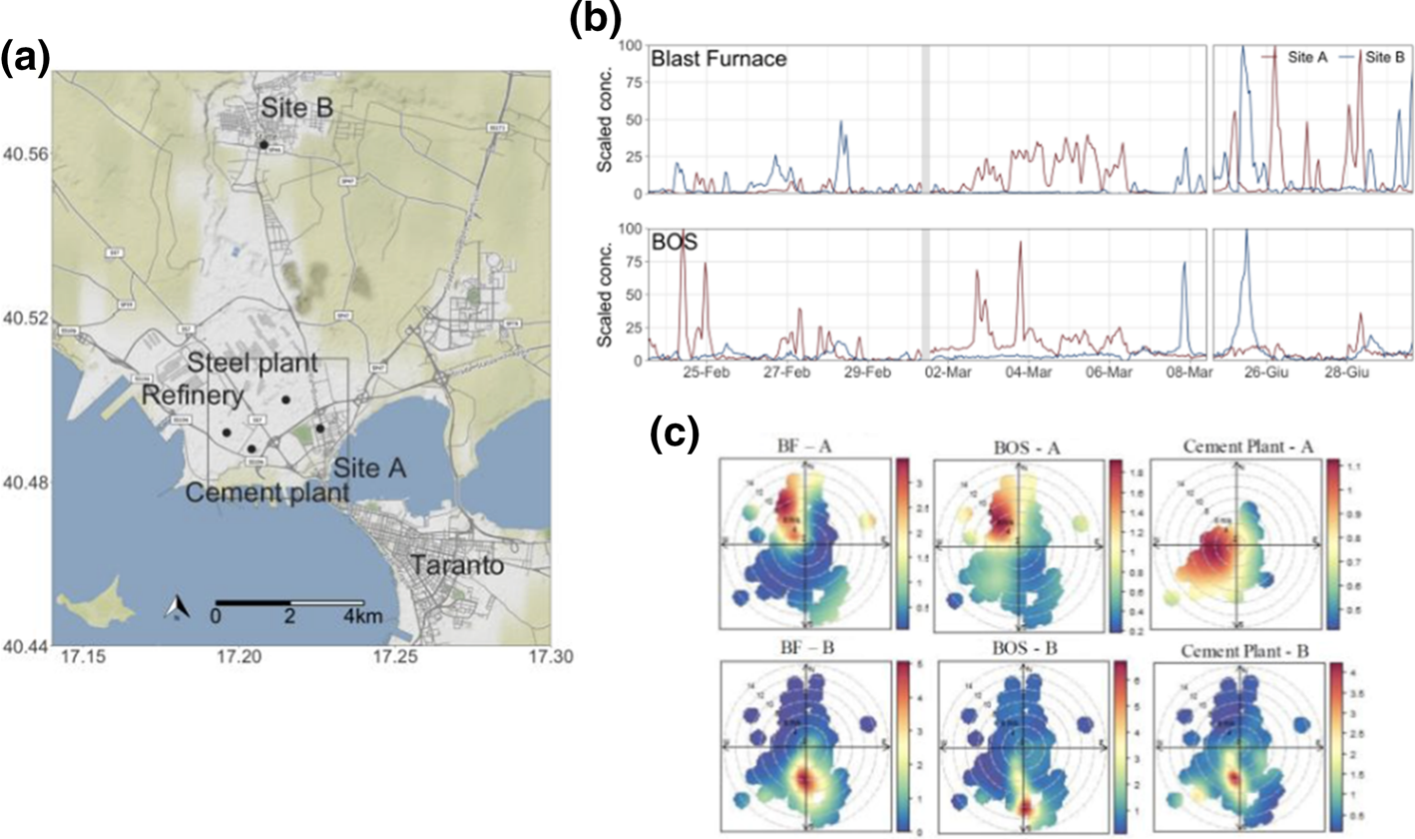
**a** Location of the industrial area and of the sampling sites on the Taranto area map. **b** The temporal patterns of two of the identified sources in the fine fraction (in arbitrary units) in both sampling sites. **c** Source polar plots as a function of wind direction and speed in site A and site B for the 3 sources identified in the fine fraction. Note the difference between the polar plots of the two sources located in the steel-making complex and the cement plant

Elemental concentration obtained with 1-h time resolution can give invaluable information for the study of episodic events, lasting a few hours that may lead to an exposure problem like the ones occurring in industrial sites. The emissions from integrated steel-making facilities are complex. The proximity of the main steelworks processes makes it difficult to distinguish individual processes. Identification is further complicated both by the influence of other external anthropogenic activities and by the simultaneous presence of continuous and discontinuous processes over time. Because of the non-continuous nature of many steel-making processes, daily filter sampling does not have sufficient time resolution to capture short-lived events arising from specific emissions. The hourly data collected at two sites in Taranto area, in southern Italy, where the biggest steel plant in Europe is located, allowed us to observe in detail the different emissions from the integrated steel-making facility (the blast furnace, the basic oxygen furnace, the sinter plant), and the impact of other industrial activities, like the cement plant. The location of the sampling sites, in opposite position with respect to the industrial site, allowed us to follow the impact of the industrial plume as a function of wind direction (see Fig. 9). Moreover, the hourly resolution demonstrated high metal concentrations lasting a few hours, which may lead to an exposure problem in this area. The presence of other anthropogenic and natural sources was also identified. The source polar plots were able to identify the directional locations of different sources identified by PMF [79].

Another possibility is the study of pyrotechnic events, such as on New Year’s Eve, national festivities and light festivals, which give rise to large (up to hundreds of μg/m^3^), but transitory (up to hours) increases of urban atmospheric particulate matter mean levels, especially metalliferous particles (K, Mg, Ba, Cu, Sr, Al, Pb) which can be dangerous for the human health. We have studied the aerosols generated by high-intensity pyrotechnic events, called mascletàs. The mascletàs take place during annual festivals in numerous cities in the Spanish Valencian region. Unlike usual fireworks, mascletàs are intended largely to stimulate the auditory system and body vibration through the strong rhythmic noise sequence produced by the burning of a type of bangers called masclets.

The masclet is a powerful sound firecracker that can be burst both at ground level and at low altitude 1.5–2.0 m). When it burns, a loud intensity detonation, light effects and abundant smoke generation are produced depending on its composition. During mascletàs, hundreds of firework shells are also shot into the air using vertical cannons. People attending these events are directly enveloped by the aerosol clouds that are produced. In this monitoring campaign the sampling site was located very close to the launching zone, so the emission aerosol cloud was measured directly. Extremely high concentrations in the fine fraction were found; maximum values above 500 μg/m^3^ for K, 100 μg/m^3^ for S and 300 μg/m^3^ for Cl were reached, with increase factors of more than 1500 compared to background levels [80]. Elements related to pyrotechnic displays like Al, Mg, Cu, Co, Zn, and Pb also showed a large rise, with increase factors above 100, mainly in the fine fraction in comparison to their normal values. In the case of Sr and Ba, factors up to 1000 were observed. Such very high concentrations in a short time in a place where thousands of people are gathered may be of concern, particularly for people who suffer from chronic respiratory health problems or cardiovascular disease.

The final example is about the high-resolution measurements of elemental composition of dust exported to the North Atlantic at Izaña Observatory (Tenerife). The aim of the study was to answer three questions: how quick does dust composition change in the Saharan Air Layer (SAL)? What is the connection to dust sources? What is the role of meteorology? A change of a factor up to 2 in the inter-elemental ratios of crustal elements was detected in only 6 h. During one week, 7 concatenated impacts, which were traced by the variability in the ratios of the different elements to Al, were observed. This variability was induced by the alternated impacts of three of the large North African dust sources. We found a correlation between dust composition in the SAL and the variability of summer meteorology. These results [81] show that long-term variability of meteorology in North Africa may have implications on the composition of the dust exported to the North Atlantic and this is relevant for the interconnection between aerosol desert dust, meteorology and climate.

### ^14^C AMS measurements on microsamples

When dealing with radiocarbon, the “natural” application one typically thinks about is its use in archaeological contexts. However, still considering topics related to history and cultural heritage, ^14^C can provide support to solve issues in most recent times as well. An example is given by artworks authenticity problems, when the chronology of materials constitutes a key point, even though not always sufficient.

**Fig. 10:**
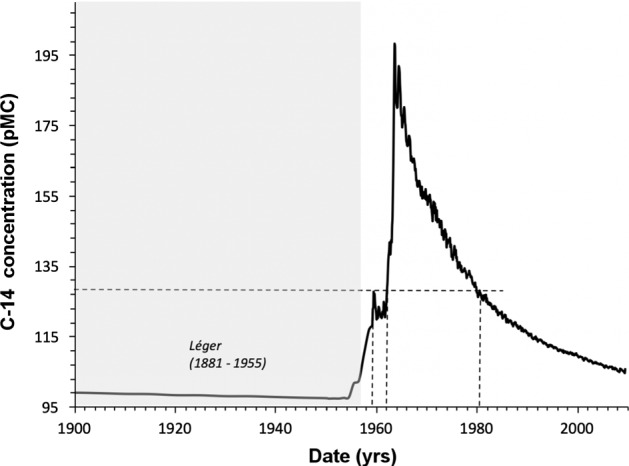
Radiocarbon measurement of the canvas sample collected from “Contraste de formes” (simplified representation of calibration); the Léger lifespan is highlighted in grey

A successful example of applying radiocarbon to the possible authentication of paintings is represented by the case of “Contraste de formes”, a painting attributed to the French painter Fernand Léger (1881–1955). This study was conducted by LABEC in collaboration with the INFN division of Ferrara and the Peggy Guggenheim Collection, Venice, to which the artwork belongs [82]. This painting was bought by Peggy Guggenheim in the early 1960s, but it was soon suspected to be a forgery and, for this reason, it has never been displayed to the public. We dated a sample collected from the rear edges of the canvas, obtaining a result that was not compatible with the Léger lifespan: indeed, the oldest period the canvas can be dated back to has resulted to be 1959, while the painter died in 1955, just before the huge increase of the ^14^C concentration in atmosphere known as Bomb Peak (see Fig. 10). In this case, when the support material is younger than the supposed artist himself, there is no ambiguity in the interpretation of the radiocarbon result. However, the “authenticity” of the support may not directly prove the authenticity of the artwork itself: in fact, expert forgers could have used an old support, contemporary with the artwork they want to make a fake of.

In general, even when authenticity is not an issue, we should always consider a possible offset between the age of the material we are dating and the “event” we are actually interested in, since a radiocarbon measurement gives us a *terminus post quem* as a result. If we date the support of an artwork, either the canvas from a painting or the paper from a written document, we may expect a possible temporal discrepancy between the age we are measuring and the manufacture of the artwork/document. A possible way to overcome this issue would be directly dating the organic fraction of pigments and/or inks. Actually, we can reasonably hypothesize that, in ancient times, inks and colours were produced in small quantities by hand using common and natural materials, so that we can generally expect a short period between their manufacture and their usage. Their dating would give us an age which is likely to be closer to the age of the event we want to date (i.e. the manufacture of the document/artwork) [83]. Such a possibility is, however, limited by the invasiveness of the measurement, considering that sampling the datable material is mandatory for a radiocarbon application, and would be feasible only when measurements with microgram-sized samples are possible. At LABEC, exploiting the new upgraded graphitization set-up and AMS procedures for very small samples, we have recently investigated the possibility to directly date organic black ink on papyrus. The choice of papyrus was given by the fact that this material has been used in many civilizations and through many different periods, as, for instance, in ancient Egypt [84, 85].

For the ink dating feasibility study, test papyrus samples were prepared, focusing on ink based on charcoal particles derived from wood combustion dispersed in Arabic gum [86], prepared following the old recipe as accurately as possible.

Considering the used materials, we expected that the datable fractions, either charcoals or the binder, would have been collected by immersing the written papyrus in warm deionized water, considering the high solubility of the Arabic gum. However, it is clear that the solubility in water of the papyrus extractives is as good as that of Arabic gum. After verifying by FTIR the basic similarities between the Arabic gum and the papyrus extractives, we decided to focus on the possibility to use the recovered charcoal particles for the ^14^C measurement.

About 0.2 ÷ 0.3 mg of charcoal particles were recovered from each of the test samples. Graphite pellets of about 50 μg of carbon each were prepared from them. In one case, the recovered material after the extraction procedure from ink was enough to prepare two graphite pellets to be measured by AMS.

**Fig. 11:**
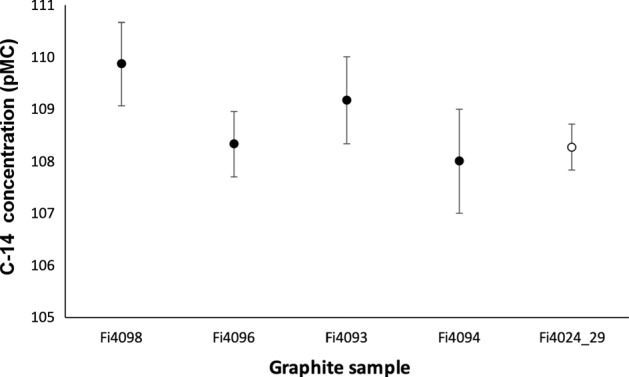
Measured radiocarbon concentrations in charcoal particles recovered from test papyrus samples (Fi4093 and Fi2094 correspond to two separate graphite pellets prepared from the same test sample); as a comparison, by open dot, the ^14^C concentration measured in the charcoal particles recovered from the ink without being used on papyrus is also reported

Measurements showed a good reproducibility among the processed samples and proved that all possible contaminants had been removed (see Fig. 11), thus suggesting that the extraction and measurement procedure can be applied to “real” ancient papyrus documents, even though in such cases we can expect that some of the original material has been lost or that a different black organic ink, produced using, for example, soot instead of wood charcoal, might have been used.

## Footnotes

1 The hill of Arcetri has been appointed as one of the “EPS Historic Sites” by the European Physical Society owing to many reasons, among which the presence there of the Villa Il Gioiello, where Galileo spent the last nine years of his life, in home confinement after the sentence of the Holy Inquisition in the 1633 trial.
